# Experimental study on the biomechanical stability of complex acetabular fractures in the quadrilateral area: application of a dynamic anterior titanium-plate screw system

**DOI:** 10.1186/s12891-024-07646-0

**Published:** 2024-07-09

**Authors:** Yong-De Wu, Xian-Zhong Mei, Wei-Fei Wu, Hong-Xi Zhang, Jie Liang, Xian-Hua Cai

**Affiliations:** 1 Department of Orthopedics, The First College of Clinical Medical Science, China Three Gorges University, Yichang Central People’s Hospital, Yichang, China; 2 Department of Orthopedics, Shenzhen Pingle Orthopedic Hospital, Shenzhen, China; 3 Department of Mechanical Room, Wuhan University of Technology, Wuhan, China; 4 Department of Orthopedics, South China Hospital of Shenzhen University, No.1 Fuxin Road, Longgang District, Shenzhen, Guangdong Province 518000 China; 5 Department of Orthopedics, General Hospital of Central Theater Command of The People’s Liberation Army, Wuhan, China

**Keywords:** Acetabular fractures, Quadrilateral area, Articular comminution, DAPSQ biomechanics

## Abstract

**Background and objective:**

Complex acetabular fractures involving quadrilateral areas are more challenging to treat during surgery. To date, there has been no ideal internal fixation for these acetabular fractures. The purpose of this study was to evaluate the biomechanical stability of complex acetabular fractures using a dynamic anterior titanium-plate screw system of the quadrilateral area (DAPSQ) by simulating the standing and sitting positions of pelvic specimens.

**Materials and methods:**

Eight formal in-preserved cadaveric pelvises aged 30–50 years were selected as the research objects. First, one hip of the normal pelvises was randomly used as the control model (group B) for measurement, and then one hip of the pelvises was randomly selected to make the fracture model in the 8 intact pelvises as the experimental model (group A) for measurement. In group A, acetabular both-column fractures in the quadrilateral area were established, and the fractures were fixed by DAPSQ. The biomechanical testing machine was used to load (simulated physiological load) from 400 N to 700 N at a 1 mm/min speed for 30 s in the vertical direction when the specimens were measured at random in simulated standing or sitting positions in groups. The horizontal displacement and longitudinal displacement of the acetabular fractures in the quadrilateral area were measured in both the standing and sitting simulations.

**Results:**

As the load increased, no dislocation or internal fixation breakage occurred during the measurements. In the standing position, the horizontal displacement of the quadrilateral area fractures in group A and group B appeared to be less than 1 mm with loads ranging from 400 N to 700 N, and there was no significant difference between group A and group B (*p* > 0.05). The longitudinal displacement appeared to be greater than 1 mm with a load of 700 mm in group A (700 N, 2 cases), and the difference was significant between group A and group B (*p* < 0.05). In the sitting position, the horizontal and longitudinal displacements of the quadrilateral areas were within 0.5 mm in group A and group B, and there was no significant difference between group A and group B (*p* > 0.05).

**Conclusion:**

For complex acetabular fractures in the quadrilateral area, DAPSQ fixation may provide early sitting stability, but it is inappropriate for patients to stand too early.

## Introduction

The term complex acetabular fracture, which involves both columns of the acetabulum, is mainly caused by high-energy injuries [1, 2]. Such fracture patterns, especially in quadrilateral areas, are more challenging to process in trauma departments of orthopedics [3, 4]. Acetabular fractures in the quadrilateral area are usually followed by multiple injuries, open anatomical reduction and internal fixation are traditional therapeutic methods, and the multiapproach operation or modified ilioinguinal approach is adopted during surgery [3, 5, 6]. However, these approaches have many drawbacks, such as nerve and artery injury, easy infection, and slow recovery. Due to complications, a single ilio-inguinal approach has been advocated [7, 8]. Nevertheless, it is difficult to fix the posterior column fracture block (quadrilateral area) through a single anterior approach because the bone in the quadrilateral area is so thin that the screws in this area can easily reach the hip [7, 9]. In recent years, several indirect fixation methods have emerged; for example, lag screws and cerclage wires may produce a certain fixed result [7, 8, 10]. Cerclage wires have been described for managing acetabular fractures in the geriatric population, but they are not commonly used in young patients [10]. However, these fixation methods require more clinical practice and biomechanical analysis, and more work is needed to identify suitable fixators [7, 11]. Wu reported the use of the dynamic anterior titanium-plate screw system of the quadrilateral area (DAPSQ) for the treatment of these fractures, and it produced good clinical effects [8]. The aim of this study was to evaluate the biomechanical stability of the DAPSQ, which would provide early sitting stability after surgery, the standing early maybe inappropriate.

## Materials and methods

### Specimens

**Fig. 1:**
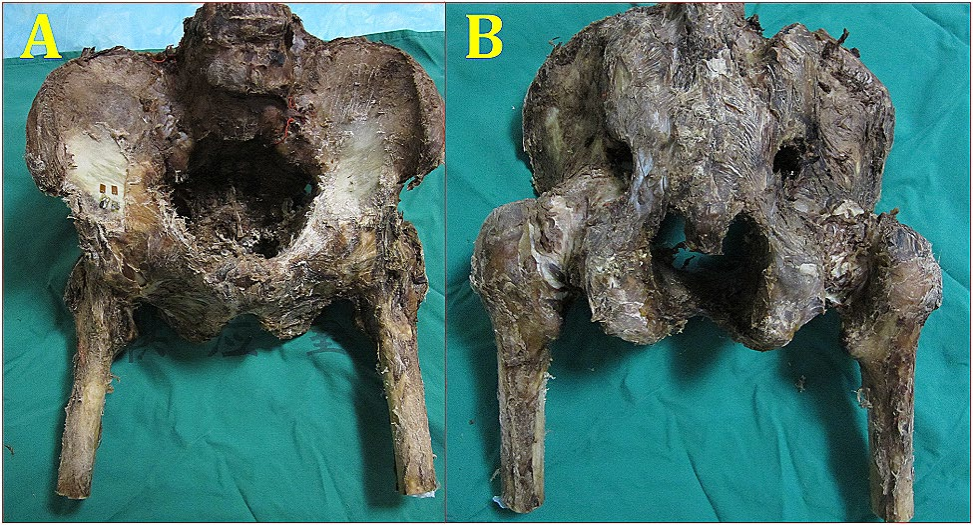
Normal specimens (1**a**: front, 1**b**: back)

**Fig. 2:**
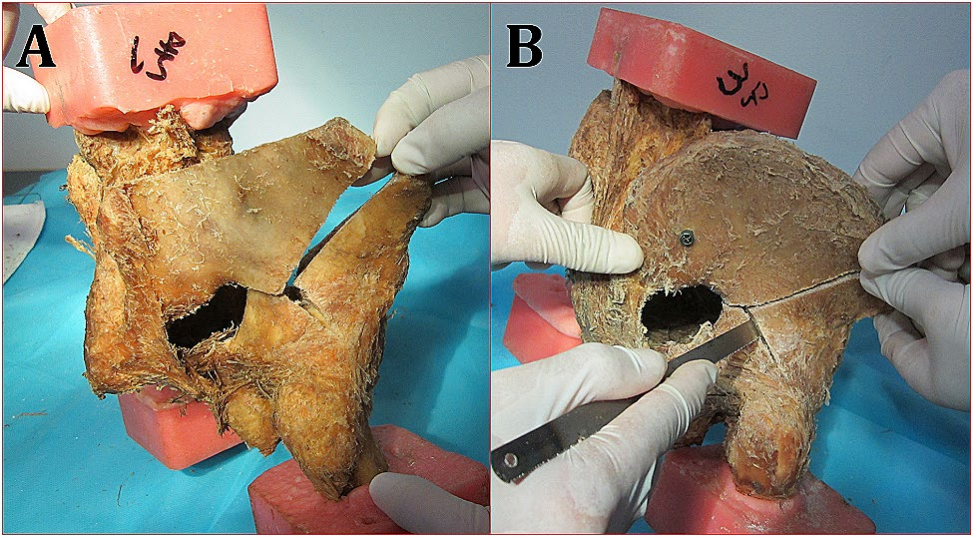
Cadaver fracture model of a high both-column fracture

This experimental study was approved by the Ethics Committee of the General Hospital of Central Theater Command of The People’s Liberation Army. In this study, eight 30- to 50-year-old formalin-preserved cadaveric pelvises were selected from Southern Medical University. The specimens with bone abnormalities were excluded by visual observation and X-ray examination. The fourth lumbar vertebra, proximal 1/3 of the femoral shaft and partial soft tissue were preserved in all specimens. To facilitate the measurement, the proximal 1/3 of the femoral shaft was solidified and fixed with denture stone (Fig. 1). First, the eight intact pelvises were used as the control models (group B) for measurement, and then, in group A, only one hip of every intact pelvis, where the model of acetabular both-column fracture was randomly established by a wire saw (Fig. 2), was used for testing after being fixed by the DAPSQ. The normal hip power in a single-leg stand is approximately 1500 N [7], we chose 700 N as the ultimate load in the bipedal standing position. After loading, the horizontal displacement of the quadrilateral area in the acetabulum and the longitudinal displacement of the acetabular fractures under 400–700 N were recorded. The anatomical reconstruction plate and titanium screws (3.5 mm) of the AO were selected for the DAPSQ. A CT machine and X-ray machine (Siemens ARCADIS ORBIC, General Hospital of Central Theater Command of The People’s Liberation Army) were used to assess the safety of the fixation. A ZWICKZ100 material machine (Germany ZWICKZ, a precision of 0.1%, Department of Mechanical Room at Wuhan University of Technology) was used to measure the displacement.

### Fixation system (DAPSQ)

**Fig. 3:**
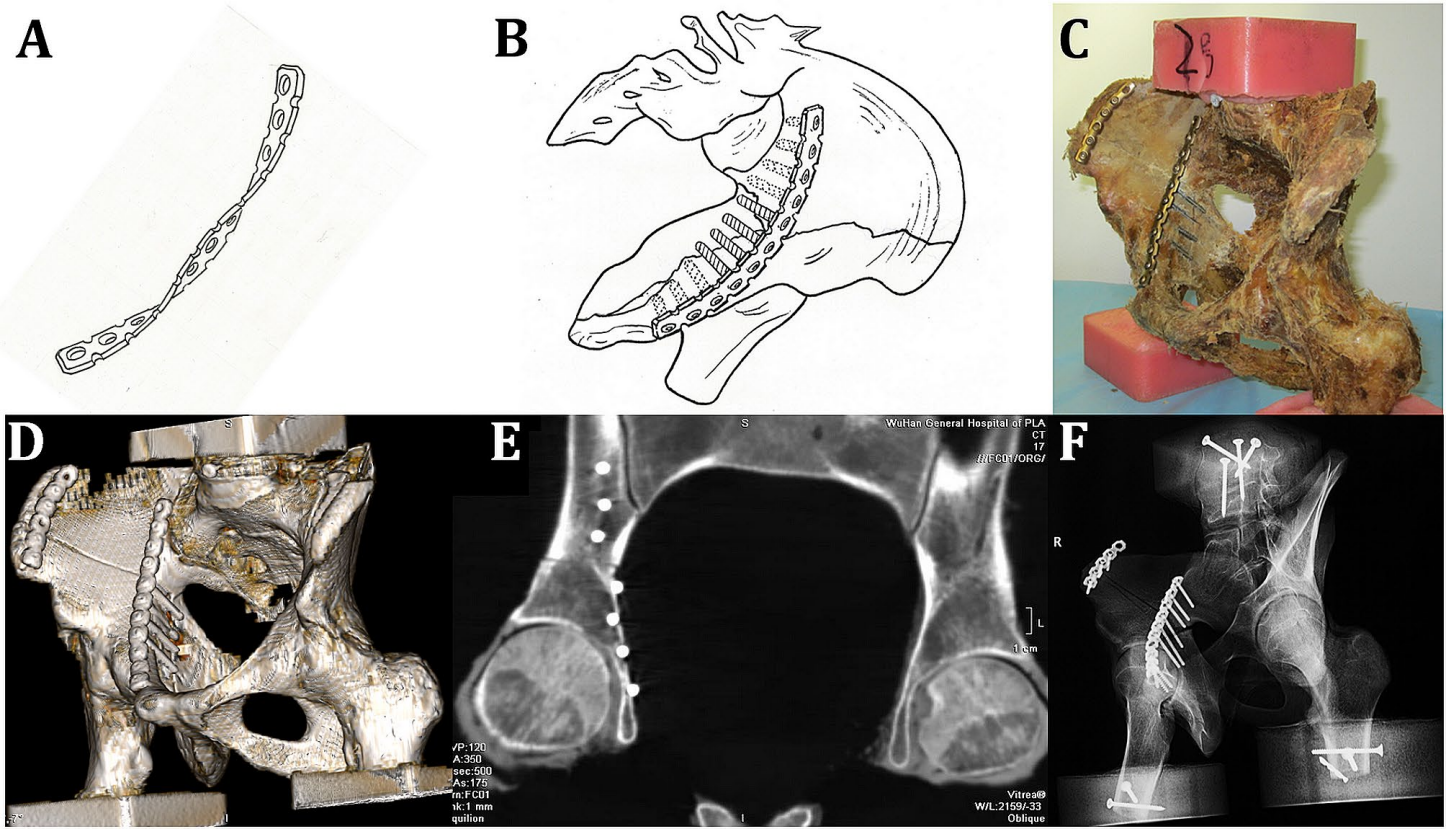
Fixation of the dynamic anterior titanium-plate screw system of the quadrilateral area (DAPSQ). (3**a**-**b**: Schematic diagram of DAPSQ, 3**c**: Photo of DAPSQ, 3**d**-**e**: CT-ray scan, 3**f**: X-ray scan of DAPSQ)

The internal fixation material used was an AO 3.5 mm reconstruction plate. The titanium plate of the DAPSQ was slightly upturned and valgus at both ends in the middle section (upper edge of the quadrilateral area) with varus. The degree of bending was approximately 15° (Fig. 3a-b). At the same time, the titanium plate was moved to the pelvic cavity at approximately 1/3 to 1/2 of the nail hole on the upper edge of the quadrilateral area, and the plate was screwed although the nail holes were parallel to the surface of the quadrilateral area. More than half of the screws were located in the bone (Fig. 3c-f). The auxiliary plate of the iliac wing fracture in the DAPSQ system shares the resistance of the acetabular anterior column plate to displacement of the fracture. Screws in the quadrilateral region of the DAPSQ system may be called semicortical screws. When screws are inserted in the quadrilateral area, the screws are not allowed to break through the inner bone plate (the side bone cortex of the hip joint) under direct observation. We can reshape steel plates, adjust the screw direction or select other screw implants. In this study, for comparative observation, 4 semicortical screws were used in each case of the acetabular area. The length of the semicortical screws should exceed the fracture line by at least 10 mm.

### Data analysis

**Fig. 4:**
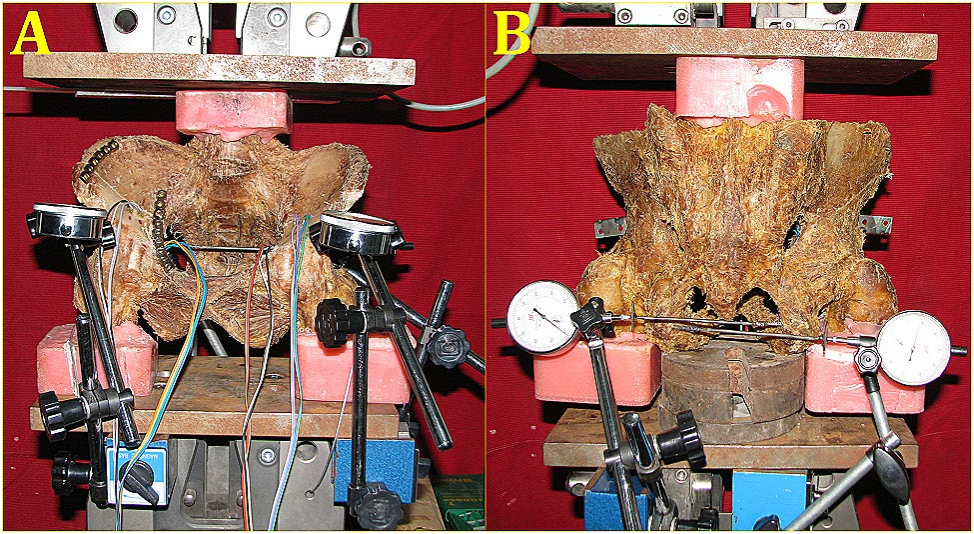
Testing in the pelvis (4**a**: standing stimulation, 4**b**: sitting stimulation)

Determination of femoral head dislocation: According to the Matta standard [12], anatomical reduction was considered when the fracture displacement did not exceed 1 mm after the operation. Therefore, central dislocation of the femoral head was considered if the lateral displacement of the inner wall of the posterior column was greater than 1 mm, and upward dislocation of the femoral head was considered if the longitudinal displacement of the anterior inferior spine was greater than 1 mm. The specimens were fixed in the position of standing and sitting simulations (Fig. 4) in the pelvis as previously described by Pierannunzii et al. and Wu et al., respectively [7, 13]. Before the test, 400 N was preloaded to eliminate the effects of bone tissue relaxation, creep, joint space and small differences in fracture reduction, etc., and then loading in the vertical direction was increased from 400 N to 700 N at a rate of 1 mm/min for 30 s. According to the simulated physiological weight load, the displacement of the acetabular fractures in the quadrilateral area under the load of 400–700 N was measured by the dial gauge of the Zwicks’ 100 electronic universal testing machine in the horizontal and longitudinal directions. The experiment was terminated when hip dislocation or internal fixation rupture occurred.

### Statistical analysis

The statistical analysis was conducted using a standard SPSS 26.0 (SPSS Institute, Chicago, IL) software package. The data are expressed as the mean ± standard deviation. The *t*-tests were performed at each of the 400 N, 500 N, 600 N, and 700 N loads to compare the significance of the differences between groups separately for each index of measurement. *P* < 0.05 was considered to indicate statistical significance, which may indicate that taking the sitting or standing position early after surgery in the DAPSQ system is unsafe.

## Results

No dislocation or internal fixation breakage occurred during the measurements. After internal fixation, the acetabular fractures in the quadrilateral area of both columns were immediately fixed, and anatomic reduction was achieved in the A group. There was no screw entering the joint cavity. Soft tissue was taught during the test process. After the removal of the load, the acetabulum and fixation returned to the initial state.

In the standing position (Tables 1 and 2), the loading-horizontal displacements from 400 N to 700 N and the loading-longitudinal displacements from 400 N to 600 N appeared to be less than 1 mm in group A, the longitudinal displacement appeared to be more than 1 mm under 700 N loading in group A (700 N2 cases), while the loading-horizontal displacements and the loading-longitudinal displacements appeared to be less than 1 mm in group B from 400 N to 700 N. The statistical analysis revealed that there was no significant difference in the horizontal displacement between group A and group B (*p* = 0.25), and there was no significant difference in the longitudinal displacement between group A and group B with loads ranging from 400 to 600 (*p* = 0.14); the difference was significant in the longitudinal displacement between group A and group B with a load of 700 (*p* = 0.01).

**Table 1:** Different loading-horizontal displacements in the standing position (${\bar x}$±s mm)

Group	Different loads (*N*)			
	400	500	600	700
A	0.26 ± 0.08	0.30 ± 0.14	0.34 ± 0.18	0.38 ± 0.23
B	0.12 ± 0.02	0.15 ± 0.03	0.18 ± 0.07	0.21 ± 0.11

Independent-sample t test: group A - group B (*p* = 0.25)

**Table 2:** Different loading-longitudinal displacements in the standing position (${\bar x}$±s mm)

Group	Different loads (*N*)			
	400	500	600	700
A	0.50 ± 0.16	0.57 ± 0.24	0.63 ± 0.25	0.81 ± 0.39
B	0.35 ± 0.14	0.42 ± 0.20	0.54 ± 0.22	0.66 ± 0.21

Independent-sample t test: group A - group B (*p* = 0.14) at each of the 400 N, 500 N and 600 N loads. group A - group B (*p* = 0.01) at the 700 N loads

In the sitting position (Tables 3 and 4), the horizontal and longitudinal displacements of the quadrilateral areas were within 0.5 mm between group A and group B. The statistical analysis showed that there was no significant difference between group A and group B (*p* > 0.05).

**Table 3:** Different loading-horizontal displacements in the sitting position (${\bar x}$±s mm)

Group	Different loads (*N*)			
	400	500	600	700
A	0.26 ± 0.08	0.30 ± 0.09	0.33 ± 0.10	0.35 ± 0.11
B	0.23 ± 0.07	0.26 ± 0.08	0.30 ± 0.13	0.33 ± 0.17

Independent-sample t test: group A - group B (*p* = 0.65)

**Table 4:** Different loading-longitudinal displacements in the sitting position (${\bar x}$±s mm)

Group	Different loads (*N*)			
	400	500	600	700
A	0.20 ± 0.13	0.24 ± 0.08	0.27 ± 0.11	0.31 ± 0.16
B	0.11 ± 0.01	0.15 ± 0.09	0.18 ± 0.30	0.21 ± 0.02

Independent-sample t test: group A - group B (*p* = 0.41)

## Discussion

The quadrilateral area was located on the inner side of the acetabular posterior column. Quadrilateral area fractures are not clearly defined in the literature, while except for simple fractures according to the classification of Letournel and Judet, the others all involve this area. It is a complex comminuted fracture caused by high-energy injuries under strong trauma from the longitudinal femoral neck. Optimal anatomical reduction and stable fixation of acetabular fractures are important for preventing secondary dislocation and osteoarthritis [14]. We often select a multiapproach operation or a modified ilioinguinal approach for exposure during the operation [12, 15]. Although ORIF has reliable stability, it has limitations in clinical application, such as significant damage due to tissue injury [16]. Single-center series suggest that 12–45% of acetabular fracture patients treated with open reduction internal fixation subsequently undergo at least one secondary surgical procedure that ablates the repaired native acetabulum [17]. Therefore, some authors have claimed that a minimally invasive percutaneous technique should be encouraged, and a single forward approach should be adopted for complex fractures of the acetabulum during surgery [18, 19]. We also found that the ilioinguinal approach provided a good view of fractures of the quadrilateral area and the partial posterior column.

The acetabulum represents a basin-like anatomic structure that bears the body’s weight and accommodates ambulatory activity and hip joint mobility [20]. The pelvic stability provided by the anterior column is 2.75 times greater than that provided by the posterior column [21]. Anatomical reduction in the anterior column combined with rigid internal fixation is the key to preserving hip function via the ilio-inguinal approach.

However, the stability of the hip mainly depends on the blocking effect of the acetabular bone. The bone in the quadrilateral area and the iliopectineal bulge of the anterior column do not enable safe implantation of screws [7, 12]. To avoid this risk, fixations involving no fixation or indirect fixation in quadrilateral areas have emerged. For example, the plate of the anterior column with no fixation in the quadrilateral area only plays a supporting role; for screws away from the acetabular fracture, the degree of fixation stability decreases by approximately 50% [7]. In recent years, drawing support from the development of imaging and navigation technology, a lag screw through the anterior column to the posterior column may produce a certain fixed result [7], but its fixation is eccentric (partial posterior), and it requires that the fracture blocks of the anterior column and posterior column are not crushed [22, 23]. The fracture may be displaced during the tightening of the interfragmentary lag screw. In addition, the cerclage wire, buttress screw or infra-pectineal plates, which are used for elastic fixation, can prevent medial displacement of the medial wall of the acetabulum (quadrilateral area) [24–26]. However, the existing hip biomechanics suggest that the bearing force of the acetabulum in the quadrilateral area can be divided into two components in the horizontal and vertical directions in the coronal plane [7]. These kinds of elastic fixation are insufficient for offsetting the vertical separation. Moreover, the stability of buttress plates has always been controversial. A clinical study showed that elastic plate stability was reduced by postoperative turning nursing care, and the plate was easily remodeled to lose its fixation role after fatigue [7, 27].

To our knowledge, there are no direct fixation types in the quadrilateral area in the previous literature, and the effect of bone block in the quadrilateral area is often neglected. Regarding how to improve the stability of the fracture block in the quadrilateral area during the operation, DAPSQ, which uses a single ilioinguinal approach to fix the bone block in the quadrilateral area under direct vision, may be a good choice. Because this is not only the typical case for fixed bone surfaces but also for a small portion of bone, it is dynamic and resistant to separation in the horizontal and vertical directions.

Clinically, how can quadrilateral area screws not enter the joint? The use of repeated intraoperative fluoroscopy or physical examination of hip motion for screening is time-consuming and less reliable. Our operation is carried out visually to avoid screws entering the joint in the DAPSQ system [8], and less intraoperative fluoroscopy is usually used for screening. When screws are inserted in the quadrilateral area, the screws are not allowed to break through the inner bone plate (the side bone cortex of the hip joint) under direct observation. If screws enter the joint, due to the use of reconstruction plates or nonlocking plates, we can reshape the steel plates, adjust the screw direction or select other screw implants. During clinical operation, 3–5 semicortical screws can be selected according to the fracture size of the acetabular area. In our clinical research [8], no screws entered the joint cavity. With the help of the DAPSQ, fractures in the medial acetabular area and the partial posterior column can be fixed perfectly via the classical ilioinguinal approach [8].

To verify the biomechanical stability of the DAPSQ, based on previous studies [7], the selected pelvic specimens were fresh anatomical specimens. All the important ligaments of the pelvic ring were preserved in the specimens. The direction of load conduction was transmitted from the vertebral body to the sacroiliac joint and the acetabulum femoral head. To fully simulate the postoperative physiological status of patients, we selected the sitting and standing positions for the experiment to explore the stability in standing or sitting position.

In group A, because the normal hip power in a single-leg stand is approximately 1500 N [7, 28], we chose 700 N as the ultimate load in the bipedal standing position. With a maximum load of 700 N in the standing position [7], the longitudinal displacement appeared to be more than 1 mm, while dislocation of the joint did not occur. This finding is not consistent with the standard proposed by Matta [13]. We suspect that soft tissue may play an important role in restoring joint stability on the DAPSQ. In fracture model designations and measurements, we found that when the fractures were not fixed, the sacrotuberous ligament and the sacrospinous ligament were relaxed, and when the fracture was reduced, especially after loading, the two ligaments recovered tension, which means that the reduction and fixation of the square fracture is closely related to the integrity of the soft tissue. When removing the obturator muscles, especially the sacrospinal ligament and sacral tuberosity ligament, in our experimental fracture model, fixation was prone to instability. We predicted that it would be inappropriate to stand too early with severe soft tissue injury.

In group A, according to measurements of the hip after loading in the simulated sitting position, no dislocation or internal fixation breakage occurred. The maximum displacements of the acetabular area fracture in the horizontal direction and vertical direction were less than 0.5 mm. According to the Matta standard [13], anatomical reduction is defined as a fracture displacement that does not exceed 1 mm after internal fixation. Perhaps we can infer that it may be safe for patients to sit early after a fracture is fixed according to the DAPSQ.

Although the experiment still offers interesting and valuable data, its limitations must be addressed. Firstly, the sample size is small, we only used eight specimens as representatives because the human pelvis specimen was very precious. Secondly, as eight specimens were tested successively firstly group B and then group A, we chose a relatively small load in this experiment to minimize interference that the changes of the pelvis after the measurement in group B maybe affect the measurement of group A. We ignore the interference to the group B in our study. Thirdly, the observational indicators are insufficient. Due to repeated measurements of specimens, we selected a relatively small limit load in measurements to ensure the integrity of the specimen. What^’^s more, the acetabular fractures in the quadrilateral area are irregular in shape, the deformation is difficult to measure. For the above reasons, the indexes such as ultimate load and stiffness could not be observed. Fourthly, we did not analyze the role of important soft tissues around the fracture. Moreover, the design control group of this study was the complete pelvis group, and no other internal fixation was used as the internal fixation control group; further comparisons are needed in the future. In addition, the fracture model is relatively simple and does not include serious acetabular fractures for extremely complex quadrilateral fractures in the clinic, especially those with obvious displacement of the posterior column or posterior wall fractures, for which unsatisfactory correction of fracture displacement after anterior approach reduction and fixation is needed to choose an additional posterior approach for fixation.

## Conclusion

This preliminary study demonstrated that for complex acetabular fractures in quadrilateral areas, the actual biomechanical effect of DAPSQ was close to that of a normal pelvis on the stability of the hip joint in sitting simulations. DAPSQ fixation may provide early sitting stability, but it is inappropriate for patients to stand too early.

## Data Availability

The datasets used and analyzed during the current study are available from the corresponding author upon reasonable request. or the Ethics Review Committee of the General Hospital of Central Theater Command of the Chinese People’s Liberation Army.
